# Budget impact assessment of Aprokam® compared with unlicensed cefuroxime for prophylaxis of post-cataract surgery endophthalmitis

**DOI:** 10.1186/s12886-015-0056-5

**Published:** 2015-07-08

**Authors:** Christine Purslow, Keith Davey, Mildred Johnson, Guilhem Pietri, Gaurav Suri

**Affiliations:** Théa Pharmaceuticals Ltd, MED IC3, Keele University Science and Business Park, Keele University, Newcastle under Lyme, North Staffordshire, ST5 5NP UK; Spire Elland Hospital, Elland Lane, Elland, West Yorkshire, HX5 9 EB UK; Moorfields Eye Hospital, 162 City Road, London, EC1V 2PD UK; Health Economic Modelling Unit, HERON™ Commercialization, PAREXEL International, Evergreen Building North, 160 Euston Road, London, NW1 2DX UK

**Keywords:** Aprokam, Cataract surgery, Cefuroxime, Cost analysis, Economic analysis, Post operative endophthalmitis

## Abstract

**Background:**

Intracameral cefuroxime is recommended as prophylaxis against postoperative endophthalmitis (POE) following cataract surgery. Aprokam is the only licensed product for prophylaxis of POE, although unlicensed intracameral cefuroxime may be administered using pre-filled syringes (PFS), either prepared in hospital by reconstituting cefuroxime via serial dilution (prepared PFS), or commercially purchased (purchased PFS). This study aimed to estimate the potential budget impact of using Aprokam over unlicensed cefuroxime for intracameral administration.

**Methods:**

A budget impact model (BIM) was developed from UK NHS hospital perspective to estimate the economic impact of adopting Aprokam compared with purchased PFS or prepared PFS for the prophylaxis of POE following cataract surgery over a 5-year time horizon. The BIM incorporated direct costs only, associated with the acquisition, delivery, storage, preparation, and administration of cefuroxime. Resource utilisation costs were also incorporated; resource utilisation was sourced from a panel survey of hospital pharmacists, surgeons, and theatre nurses who are involved in the delivery, storage, preparation, quality assurance, or administration of cefuroxime formulations. Unit costs were sourced from NHS sources; drug acquisition costs were sourced from BNF. The model base case used a hypothetical cohort comprising of 1000 surgeries in the first year and followed a 5.2 % annual increase each year.

**Results:**

The model predicts Aprokam is cost saving compared with purchased PFS, with a modest increase compared prepared PFS over 5 years. There are total savings of £3490 with Aprokam compared with purchased PFS, driven by savings in staff costs that offset greater drug acquisition costs. Compared with prepared PFS, there are greater drug acquisition costs which drive an increased total cost over 5 years of £13,177 with Aprokam, although there are substantial savings in staff costs as well as consumables and equipment costs.

**Conclusions:**

The lower direct costs of using Aprokam compared with purchased PFS presents a strong argument for the adoption of Aprokam where purchased PFS is administered. The additional benefits of Aprokam include increased liability coverage and possible reduction in dilution errors and contaminations; as such, in hospitals where unlicensed prepared PFS is used, modest additional resources should be allocated to adoption of Aprokam.

## Background

Postoperative endophthalmitis (POE) is defined as an inflammation of the eye arising from a bacterial, fungal, or parasitic infection during the postoperative period of surgery [1]. This infection can originate from either a patient’s own flora or from contaminated surgical instruments [2, 3]. POE is associated with adverse clinical outcomes that include reduced vision and severe pain [1]. In the United Kingdom (UK), the annual incidence of POE following cataract surgery is estimated at 0.14 % [4].

In 2013, the European Society of Cataract and Refractive Surgery (ESCRS) updated guidelines that recommended use of intracameral cefuroxime as prophylaxis against POE following cataract surgery [1]. This was based on an ESCRS study conducted in nine European countries including the UK (September 2003–January 2006), which demonstrated that use of intracameral cefuroxime as prophylaxis against POE resulted in approximately a seven-fold reduction in the incidence of POE compared with placebo (from 0.345 % to 0.049 %) [5]. This recommendation is shared by the Royal College of Ophthalmologists [6] and is also supported by a number of retrospective studies [1].

Despite the ECSRS recommendations, use of intracameral cefuroxime has not been fully adopted. An ESCRS survey of member ophthalmic surgeons conducted in 2012 reported that the most common reasons for not using intracameral cefuroxime during cataract surgery were: the unavailability of the country/clinic specific protocol, unavailability of an approved preparation, and concern over the risk of dilution errors [7]. These major concerns are directly addressed by Aprokam®; which, as per the UK National Health Service (NHS) National Patient Safety Agency (NPSA) injectable risk assessment proforma, is likely to be classified as a lower-risk product [8]. Aprokam is available in 50 mg vials comprising of cefuroxime that is reconstituted via a simple one-step process to a solution for intracameral injection of 1 mg cefuroxime constituting of 0.1 ml volume of saline solution [7, 9]. Aprokam is currently the only licensed product for POE prophylaxis, and received approval by the European Medicines Agency (EMA) in 2012 [9].

Prior to the availability of Aprokam, unlicensed formulations of intracameral cefuroxime have been administered using pre-filled syringes of cefuroxime (PFS) from a variety of sources. Firstly, PFS is prepared in theatre by serial dilution of 250 mg (or larger) vials of cefuroxime licensed for injection or infusion. Secondly, some hospital pharmacy departments prepare 10 mg/ml PFS (1 mg/0.1 ml) (by serial dilution as above, but in a hospital pharmacy/sterile supplies/pharmacy manufacturing unit [pmu] as opposed to in theatre), and lastly, PFS are commercially available from a variety of pharmaceutical sources.

Although the availability of a licensed alternative should discourage use of these unlicensed formulations, the use of PFS is still practiced. The protocol for the preparation of PFS at the hospital is complex and varies among hospitals [10, 11]. Preparation of sterile preparations (such as PFS) should be carried out by trained staff (i.e. theatre nurse or pharmacy technician) in a clean environment (i.e. using a laminar hood or a glove box isolator) to ensure quality, sterility, and safety of the preparation [12]. Non-adherence to protocol [10] or inadequate mixing [13] of cefuroxime in the syringe, which are likely with the unlicensed preparation, can result in dilution errors and contamination of purchased or prepared PFS. Dilution errors can increase the dose of cefuroxime substantially above the recommended 1 mg dose, with potential clinical implications [11, 14, 15]. Cefuroxime concentration above 2.75 mg/mL has been shown to reduce the viability of human endothelial corneal cells [16].

Furthermore, serious macular detachment, macular infarction, macular oedema, corneal oedema, and haemorrhagic retinal Infarction are among the potential outcomes arising from the administration of intracameral cefuroxime at doses that are higher than recommended [10, 11, 14, 15, 17]. Contamination is another serious consideration, with incidences of POE following cataract surgery caused by Fusarium species from contaminated cefuroxime reported in the literature [18]. Unlicensed cefuroxime formulations may not provide adequate liability coverage compared with Aprokam, with ESCRS guidelines stating that, there should be awareness of the implications surrounding liability when using unlicensed cefuroxime injectable formulations [1].

The preparation of intracameral cefuroxime of any type (Aprokam or PFS) incurs costs associated with staff, consumables, equipment, and quality assurance. Whilst the clinical arguments (lower risk of dilution errors) supporting Aprokam over PFS are unequivocal, there remains some reluctance among budget-holders to adopt the licensed product available after consideration of the relatively higher acquisition costs associated with Aprokam. However, there may be considerable cost savings associated with staff time, consumables, and/or equipment with Aprokam. There may also be additional costs associated with the rare, but serious adverse clinical events that might result from unlicensed cefuroxime use, as well as reduced liability coverage for the hospital, both of which are difficult to evaluate.

In addition to the clinical arguments, economic analyses have demonstrated the cost-effectiveness of cefuroxime compared with other antibiotics in preventing POE following cataract surgery, further validating the use of cefuroxime [19, 20]. However, no studies have been reported that compare the cost differences of the variety of cefuroxime injectable formulations available. One economic analysis of Aprokam to date, conducted in a French private hospital, reported that the use of Aprokam in cataract surgeries demonstrated cost savings with improved clinical outcomes compared with cataract operations without using cefuroxime [21].

The objective of this study was to develop a budget impact model (BIM) to estimate the potential budget impact of a UK National Health Service (NHS) hospital adopting Aprokam in place of unlicensed cefuroxime formulations (prepared PFS or purchased PFS) for the prophylaxis of POE following cataract surgery. The model estimated costs over a 5-year time horizon. Clinical outcomes were not considered in this study and all cefuroxime formulations were assumed to have the same efficacy and safety. The results of this study are of use for NHS managers, payers, and clinicians, who can consider the results of this analysis in the wider context of cefuroxime use, the license status and risk of dilution errors of each cefuroxime formulation, when making decisions upon the introduction of Aprokam in a clinical setting.

## Methods

An economic model was developed in Microsoft Excel to assess the potential budget impact of using Aprokam compared with purchased PFS or prepared PFS for the prophylaxis of POE following cataract surgery. Budget impact was estimated over a 5-year time horizon (base year 2013), from the perspective of a UK NHS hospital setting. Since all three formulations are based on the same active compound (cefuroxime), their efficacy and safety profiles were assumed to be equal.

### Modelling approach overview

The BIM incorporated direct costs only, associated with the acquisition, delivery, storage, preparation, and administration of cefuroxime. The model took into account the annual number of cataract surgeries to estimate the total costs related to the prophylaxis of post-cataract surgery POE. The analysis was carried out for a hypothetical cohort comprising of 1000 surgeries in the starting year and followed by a 5.2 % annual increase. The annual increase in the number of cataract surgeries was based upon NHS data between 1998 and 2009 [6].

The model accounted for resources required to perform quality assurance during the preparation of PFS at the hospital pharmacy (members of staff, consumables, and equipment) as per the quality assurance protocols for compounding sterile preparations published by the Royal Pharmaceutical Society in the UK [22], the World Health Organization [23], and the American Society of Health-System Pharmacists [12]. These protocols recommend ingredient identity, strength, sterility, purity and environmental tests to be conducted. Quality assurance was also considered at delivery (e.g. checking the labels of purchased PFS) and during the storage of the products (e.g. temperature control).

Cost components were estimated based on resource utilisation and resource unit costs (in 2013 GB £). Resource utilisation inputs were sourced from a panel survey submitted to hospital pharmacists, surgeons, and theatre nurses who are involved in the delivery, storage, preparation, quality assurance, or administration of cefuroxime formulations used as a prophylaxis treatment against POE. Unit costs were sourced from targeted literature searches using publicly available sources.

#### Acquisition cost of cefuroxime

Annual acquisition costs were estimated for the number of Aprokam vials, 750 mg vials of Zinacef® (powdered cefuroxime), or ready-to-use PFS purchased each year by the hospital, and their unit costs. The annual number of Aprokam vials and ready-to-use PFS of cefuroxime purchased by the hospital were calculated from the number of cataract operations performed. The number of vials of powdered cefuroxime purchased was based on the number of cataract operations, but also the number of individual doses prepared with each vial, and the number of individual preparations used for quality assurance purposes.

#### Cost of staff

Cost of members of staff involved was based on their hourly wages and their working time related to the ordering, delivery, storage, preparation, quality assurance, or administration of cefuroxime. Their hourly wages were sourced from the annual salaries published by the NHS, to which the methods for hourly wage calculations published by the Personal Social Services Research Unit were applied.

#### Cost of consumables

These costs were related to the provision of consumables required for the preparation or the administration of intracameral cefuroxime, for example syringes, syringe caps, needles, filters, connectors, sterile bags, 0.9 % NaCl solution, heat-seal sterile pouches, and sample tubes. Their unit costs were sourced from various UK suppliers.

#### Cost of equipment

These costs were related to storage equipment (e.g. pharmacy fridge), preparation equipment (e.g. beta-lactam laminar hood), and quality assurance equipment (e.g. pH meter, osmometer, monochromator). The model only accounted for the proportion of the equipment value specifically dedicated to the delivery, storage, preparation, or quality assurance of cefuroxime, as informed by the panel survey. The corresponding value of equipment was then extrapolated over a 5-year depreciation time period, as per the guidelines of the finance manual issued by the UK Department of Health [24]. Additionally, an annual maintenance cost corresponding to 12 % of the equipment value was applied [25]. Acquisition costs of equipment were sourced from various UK suppliers.

### Model settings

Base case values for the model settings are shown in Table 1.

**Table 1:** Base case settings of the BIM

Variables	Details
Country and perspective	United Kingdom; NHS and PSS perspective
Currency	GB 2013 £
Time horizon	5 years
Intervention	Aprokam vials of 50 mg, reconstituted into one single 1 mg/0.1 mL individual dose administered intracamerally by slow injection into the anterior chamber of the eye [9]. Resource utilisation inputs used in the model for the reconstitution of Aprokam follow the Aprokam SPC guidelines [9]
Comparators	PFS of cefuroxime prepared in the pharmacy hospital, using Zinacef 750 mg vials
	Refrigerated ready-to-use PFS purchased by the hospital from manufacturers
Number of cataract surgeries in first year	Hypothetical cohort of 1000 cataract surgeries performed in the first year
Annual increase in cataract surgeries	Annual increase of 5.2 % in the number of cataract surgeries. This is based on the average annual increase in the number of cataract surgeries performed within the NHS in England between 1998 and 2009, as reported by The Royal College of Ophthalmologists [6]

*BIM*: budget-impact model; *GB*: Great Britain; *NHS*: National Health Service; *PFS*: pre-filled syringes; *PSS*: Personal Social Services; *SPC*: Summary of Product Characteristics

### Drug-acquisition costs

Drug-acquisition costs were based on the unit costs of Aprokam, PFS syringes (for purchased PFS), and Zinacef (for prepared PFS), as published in the British National Formulary (Table 2).

**Table 2:** Drug acquisition costs of Aprokam, purchased PFS, and prepared PFS (2013 GB £)

	Aprokam	Purchased PFS	Prepared PFS
Formulation	Vial	Syringe	Vial (Zinacef)
Dose	50 mg	1 mg/0.1 mL	750 mg
Number of individual doses per unit	1	1	26
Unit cost (2013 GB £)	7.95 [28]	7.00 [25]	2.34 [28]

*BIM*: budget-impact model; *GB*: Great British; *PFS*: pre-filled syringes; *UK*: United Kingdom

### Model inputs

Cost inputs, presented in Table 3, show resource utilisation and unit costs used in the model.

**Table 3:** Summary of resource utilization and unit cost/annual cost for each cefuroxime formulation (2013 GB £)

	Resource utilization (number of units or staff time in minutes)			Unit cost/annual cost (2013 GB £)	Comments
	Aprokam	Purchased PFS	Prepared PFS		
Delivery					
Pharmacy store manager (delivery)	2.5	1.5	2.5	45.8 [29, 30]	Time related to the paperwork upon delivery
Nurse team manager	9.9	43.0	18.0	50.0 [30]	Time related to weekly (prepared and purchased PFS) or monthly (Aprokam) ordering of individual doses by the theatre nurse to check the theatre stock (3 min), order additional stock to the hospital pharmacy and store it (15 min) and order additional stock from the manufacturer directly (purchased PFS and Aprokam only, 25 min)
Pharmacy store manager (QA during delivery)	N/A	1.3	N/A	45.8 [29, 30]	Time spent to check labelling of purchased PFS
Pharmacy store manager (transfer to storage area)	1.7	1.7	1.7	45.8 [29, 30]	Time related to the transport from delivery area to storage pharmacy storage
Storage					
Pharmacy fridge	N/A	1	N/A	132.0 [31]	Upon delivery, only refrigerated PFS requires storage in a fridge
Operating room fridge	N/A	1	N/A	37.7 [32]	
Porter (transport between storage areas)	9.6	9.6	9.6	20.0 [29, 30, 33]	Time spent to transport cefuroxime from pharmacy to theatre
Pharmacy technician (QA)	N/A	0.5	N/A	45.8 [29, 30]	Daily time spent for temperature control
Theatre nurse (QA)	N/A	0.5	N/A	43.6 [29, 30]	Daily time spent for temperature control
Preparation					
Consumables					
1 ml syringe	1	N/A	27	0.11 [34]	The list of consumables required for the reconstitution of Aprokam is based on the reconstitution protocol described in Aprokam SPC related to one single dose
10 ml syringe	N/A	N/A	1	0.17 [34]	The list of consumables used for the preparation of PFS at the hospital pharmacy is based on feedback from the panel survey. The list of consumables also includes those related to quality assurance procedures
15 ml syringe	1	N/A	N/A	0.20 [34]	
					The model assumes that 15 mL of saline solution are required for the preparation of one individual PFS
50 ml syringe	N/A	N/A	2	0.56 [34]	
Drawing up needle	2	N/A	2	0.02 [35]	
0.2 μm filter	N/A	N/A	1	2.31 [34]	
Connector	N/A	N/A	3	1.09 [36]	
Sterile syringe cap	N/A	N/A	27	1.24 [37]	
1 ml 0.9 % NaCl solution	5	N/A	408	0.01 [38]	
Heat-seal sterile pouch	N/A	N/A	52	0.07 [39]	
Equipment					
Laminar hood	N/A	N/A	1	655.1 [40]	Injectable syringes of cefuroxime are required to be prepared in a controlled atmosphere
Pharmacy fridge	N/A	N/A	1	132.0 [31]	During transfer to storage, storage, storage control and any QA test performed during storage, only prepared PFS requires. storage in a pharmacy fridge
Operating room fridge	1	1	1	37.7 [32]	During transfer to storage, storage, storage control and any QA test performed during storage, all cefuroxime formulations requires storage in an operating room fridge
Staff					
Pharmacy technician (preparation)	N/A	N/A	36.0	45.8 [29, 30]	Time spent to perform the preparation of PFS in the pharmacy
Theatre nurse (transfer to storage)	N/A	1.0	N/A	43.6 [29, 30]	Time spent to transfer purchased PFS to storage area
Pharmacy technician (storage)	N/A	N/A	13.1	45.8 [29, 30]	Time spent post-preparation to transfer to storage, conduct, storage control and any other QA test performed during storage
Theatre nurse (preparation)	2.3	1.5	N/A	43.6 [29, 30]	Time spent in the theatre for the reconstitution of Aprokam or the preparation of purchased PFS
QA of prepared PFS					
HPLC diode array detector (QA - Ingredient identity test)	N/A	N/A	1	46.9 [41]	The list of these resources was based on the quality assurance protocols for compounding sterile preparations published by the Royal Pharmaceutical Society in the UK [22], the World Health Organization [23], and the American Society of Health-System Pharmacists [12], to prevent dilution errors and contamination
Monochromator (QA - Ingredient identity test)	N/A	N/A	1	34.6 [42]	
UV-vis spectrophotometer (QA - Ingredient identity test)	N/A	N/A	1	168.0 [43]	
Head pharmacist (QA - Ingredient identity test)	N/A	N/A	2.5	49.4 [29, 30]	
HPLC diode array detector (QA - Strength test)	N/A	N/A	1	46.9^*^ [41]	
Head pharmacist (QA - Strength test)	N/A	N/A	2.5	49.4 [29, 30]	
Sample tube (QA- Purity test)	N/A	N/A	1	0.26 [44]	
pH meter (QA- Purity test)	N/A	N/A	1	12.3 [45]	
Osmometer (QA- Purity test)	N/A	N/A	1	73.7 [46]	
Head pharmacist (QA - Purity test)	N/A	N/A	2.5	49.4 [29, 30]	
Incubator (QA - Sterility test)	N/A	N/A	1	153.1 [47]	
Head pharmacist (QA - Sterility test)	N/A	N/A	2.5	49.4 [29, 30]	
Head pharmacist (QA - Environmental test)	N/A	N/A	2.5	49.4 [29, 30]	
Head pharmacist (QA - Label and bagging)	N/A	N/A	2.1	49.4 [29, 30]	
Administration					
Surgeon	1.0	1.0	1.0	100.0 [29, 30]	Time spent to intracamerally administer cefuroxime
Theatre nurse (QA)	N/A	N/A	1.0	43.6 [29, 30]	Time spent to check prior to intracameral administration

Table legend: Cost inputs by phase (delivery, storage, preparation, and administration) of the cefuroxime preparation. Cost inputs are represented by resource utilisation (number of units or staff time in minutes) and the cost of the unit/annual cost of the equipment. Resource utilisations for equipment and consumables inputs presented here are rounded to the nearest whole number. Furthermore, cost inputs are constituted by consumables, equipment, and staff. Information pertaining to the cost inputs is presented in the right hand column of the corresponding cost inputs

*GB*: Great Britain; *HPLC*: high performance liquid chromatography; *NaCl*; sodium chloride; *N/A*: not applicable; *PFS*: pre-filled syringes; *QA*: quality assurance; *UV-vis*: ultraviolet-visible spectroscopy

### Model assumptions

Based on the panel survey, the model assumed weekly deliveries of cefuroxime products and the preparation of PFS by pharmacy technicians in weekly batches using 750 mg vials. Quality assurance protocols for PFS prepared at the hospital pharmacy are necessary to prevent dilution errors and contamination and every hospital is responsible for ensuring these protocols are in place. The model therefore considered a default quality assurance protocol based on the guidelines for compounding sterile preparations published by the Royal Pharmaceutical Society in the UK [22], the World Health Organization [23], and the American Society of Health-System Pharmacists [12]. Each step of the quality assurance process requires a number of individual doses to be tested, and the model assumed that altogether 7 individual doses of prepared PFS are necessary to conduct quality assurance tests during the preparation of a weekly batch: one for the ingredient identity test, one for the strength test, one for the purity test, and four for the sterility test. These individual doses were then discarded. As a result, the estimated number of individual doses prepared with each Zinacef vial was 26. As per the Aprokam Summary of Product Characteristics (SPC) recommendations, the model assumed that one single dose is reconstituted per vial.

Since all three products contain the same active molecule, the model assumed the same efficacy between Aprokam and the comparators. Other outcomes may differ between the three products, such as the risks of complications related to preparation errors, needle stick injuries and wastage. However, due to lack of data, the model assumed these risks to be negligible for all three products.

## Results

### Aprokam vs. purchased PFS

The base case analysis showed that when Aprokam replaced purchased PFS, the overall total cost over 5 years was reduced by 4.8 % (total cost: £69,052 with Aprokam and £72,541 with purchased PFS) (Table 4). Total cost per cataract surgery was estimated at £12.5 with Aprokam and £13.1 with purchased PFS (Table 4). This corresponded to total cost savings with the adoption of Aprokam in place of purchased PFS of £3490 over 5 years and £0.6 per cataract surgery (Table 4).

**Table 4:** Costs of Aprokam compared with purchased PFS (2013 GB £)

	Aprokam	Purchased PFS	Difference
Overall total costs over 5 years (2013 GB £)	69,052	72,541	−3490
Total costs per cataract surgery (2013 GB £)	12.5	13.1	−0.6

*GB*: Great Britain; *PFS*: pre-filled syringes

**Fig. 1:**
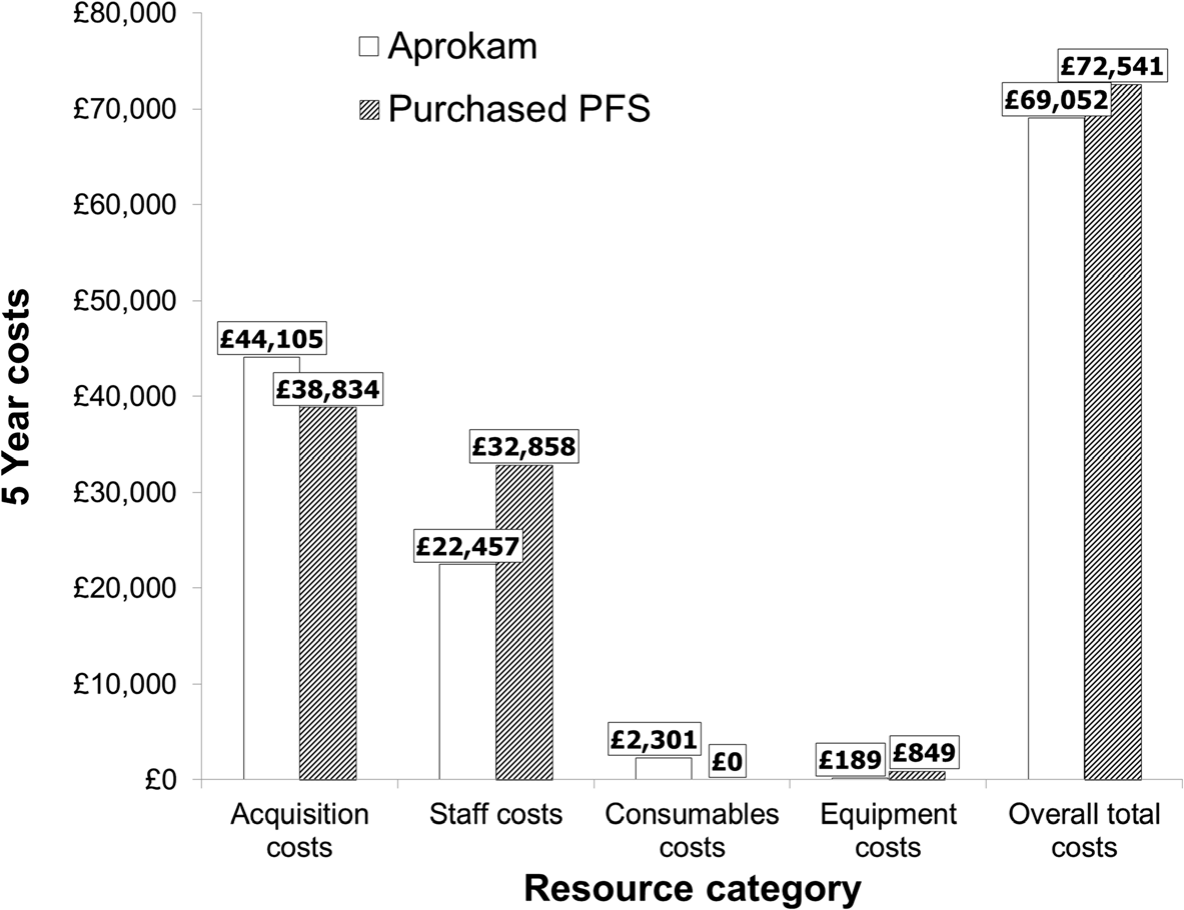
Base cast total cost of Aprokam and purchased PFS by resource category over 5 years (2013 GB £). GB: Great Britain; PFS: Pre-filled syringes

The costs of both treatments were reported by resource category. The key driver for total cost savings with Aprokam over 5 years was staff costs; with savings of £10,401 compared with purchased PFS (Fig. 1). This saving offsets the increase in acquisition and consumables costs with Aprokam (Fig. 1). Staff costs comprised of just 32.5 % of total costs with Aprokam, compared with 45.3 % of the total costs with purchased PFS. Acquisition costs represented more than half the total costs for both treatments, although were responsible for a greater proportion of the overall cost for Aprokam (63.9 %) compared with purchased PFS (53.5 %) (Fig. 1).

### Aprokam vs. prepared PFS

The base case analysis revealed that when Aprokam replaced prepared PFS, the overall total cost over 5 years was increased by 23.6 % (total cost: £69,052 with Aprokam and £55,875 with prepared PFS) (Table 5). Total cost per cataract surgery was estimated at £12.5 with Aprokam and £10.1 with prepared PFS (Table 5). This corresponded to an incremental budget impact of £13,177 and an additional cost of £2.4 per cataract surgery with Aprokam over the 5 years compared with prepared PFS (Table 5).

**Table 5:** Costs of Aprokam compared with prepared PFS (2013 GB £)

	Aprokam	Prepared PFS	Difference
Overall total costs over 5 years (2013 GB £)	69,052	55,875	13,177
Total costs per cataract surgery (2013 GB £)	12.5	10.1	2.4

*GB*: Great Britain; *PFS*: pre-filled syringes

**Fig. 2:**
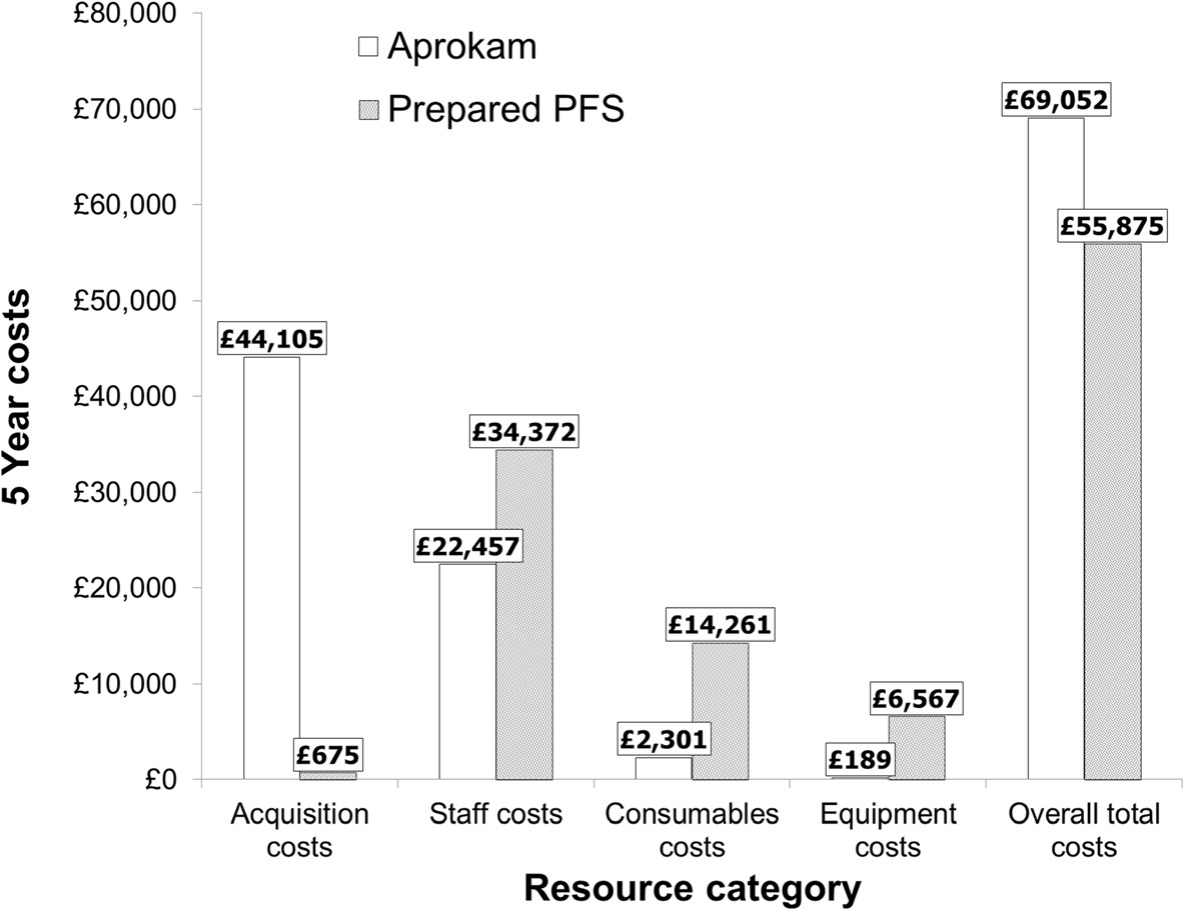
Base cast total cost of Aprokam and prepared PFS by resource category over 5 years (2013 GB £). GB: Great Britain; PFS: Pre-filled syringes

By resource category, the key driver for the cost increase with Aprokam over 5 years was acquisition costs; a budget impact of £43,430 was predicted compared with acquisition costs incurred with prepared PFS (Fig. 2). Higher acquisition costs with Aprokam were not compensated by cost savings predicted over prepared PFS in the other resource categories (consumables, equipment, and staff) (Fig. 2). Staff costs represented 61.5 % of the total costs associated with prepared PFS and 32.5 % of the total costs with Aprokam, leading to cost savings of £11,915 with Aprokam (Fig. 2). Consumables and equipment were 6.2 times and 34.8 times more expensive with prepared PFS over Aprokam, respectively, corresponding to cost savings with Aprokam in these resource categories (Fig. 2).

## Discussion

To our knowledge, this is the first published study to estimate the budget impact of introducing Aprokam in place of unlicensed PFS cefuroxime formulations that are either prepared at the hospital pharmacy or purchased as a ready-to-use formulation for the prophylaxis of POE following cataract surgery. This analysis was developed from a UK NHS hospital perspective to assist clinicians, managers, and payers in making decisions about adopting Aprokam. The outputs of the model provided an estimate of the direct economic consequences of adopting Aprokam compared with two unlicensed formations of intracameral cefuroxime.

This model estimated the budget impact of using Aprokam over a 5-year time horizon for a cohort of 1000 surgeries in the first year and assuming a 5.2 % annual increase in cataract surgeries. The results showed that Aprokam was a cost-saving alternative to purchased PFS. Use of Aprokam was predicted to deliver total cost savings of £0.6 per cataract surgery over purchased PFS, corresponding to a reduction in total costs of 4.8 %. Aprokam, however, was found in this analysis to have 23.6 % higher overall total costs when compared with PFS prepared locally at the hospital pharmacy.

Cost savings with Aprokam compared with purchased PFS were primarily driven by staff costs. The availability of Aprokam as a ready to prepare formulation allows hospitals to stock long-term supplies without product wastage. Results from pharmacy managers in the panel survey suggest purchased PFS would need to be ordered in at least weekly to ensure there is a ready supply of PFS each week. Aprokam accrued lower storage costs compared with purchased PFS, as a consequence of the refrigeration required with purchased PFS. In addition to the extra equipment (pharmacy fridge and operating room fridge) costs, there are costs associated with resource utilisation for transfers between storage areas and surgery. Furthermore, there are also costs associated with quality assurance steps that should be undertaken with purchased PFS during delivery and storage, unlike with Aprokam.

While there were extra costs related to the reconstitution of Aprokam as opposed to ready-to-use purchased PFS which do not require any preparation (e.g. extra staff time such as theatre nurse) and consumables (syringes, drawing up needle, and 1 ml 0.9 % NaCl solution), the overall staff costs remained substantially reduced.

In contrast, compared with prepared PFS, an incremental budget impact of £13,177 over 5 years was estimated with Aprokam. The key cost driver responsible for the higher total cost was greater costs incurred with the acquisition of Aprokam. These acquisition costs were estimated to be over 65 times higher with Aprokam compared with prepared PFS. Preparation of PFS in the hospital pharmacy is a complex procedure and requires numerous steps as well as high amounts of consumables, and specialised equipment (e.g. the use of a laminar hood in order to ensure a controlled atmosphere for the preparation of PFS cefuroxime).

Additionally, the risk of dilution errors associated with prepared PFS [13] necessitates adherence to quality assurance protocol. Published protocols recommend ingredient identity, strength, sterility, purity, and environmental tests to be performed for prepared PFS [12, 22, 23]. Such protocols are not required for Aprokam. As a result, preparation of PFS within the hospital requires extra consumables (sample tube), equipment (high performance liquid chromatography [HPLC] diode array detector, and monochromator, ultraviolet-visible [UV-vis] spectrophotometer, pH meter, osmometer, incubator), as well as pharmacist and nurse time. In addition, individual preparations of cefuroxime used during quality assurance are discarded and cannot be administered.

The economic impact of adopting Aprokam is just one of a number of factors that should be used to guide decision-making at the hospital. ESCRS guidelines recommend intracameral cefuroxime for the prophylaxis of POE for patients undergoing cataract surgery [1]. Further, Aprokam is currently the only licensed intracameral cefuroxime formulation [1]. Use of unlicensed cefuroxime places prescribers and the hospital with greater responsibility in terms of liability as a result of harm to the patient, compared with the use of licensed Aprokam [26].

There are also patient safety considerations to take into account when selecting cefuroxime formulations. Unlicensed formulations of intracameral cefuroxime appear to be associated with a high risk of dilution error – both over- and under-dosing [13]. Dilution errors may occur as a consequence of the multi-step dilution process and overdosing up to 50 times the recommended concentration has been reported, leading to postoperative inflammation of the anterior chamber, corneal oedema, elevated intraocular pressure, and cystoid macular oedema [10, 17]. Doses of intracameral cefuroxime lower than recommended for POE prophylaxis can also result from dilution errors with unlicensed formulations [13]. Such suboptimal dosage can potentially lead to breakthrough POE or promote the development bacterial resistance [27].

When considering whether to adopt Aprokam, decision makers such as clinicians, NHS managers, and local payers should consider the liability status of unlicensed cefuroxime formulations and the higher probability of dilution errors with prepared PFS as well as the economic considerations. Unlicensed cefuroxime places the patient at risk in terms of increased likelihood of receiving high doses that can result in complications such as macular oedema. Furthermore, there is risk to the hospital with the use of unlicensed treatments in terms of reduced liability coverage. The lower estimated total costs with Aprokam compared with PFS purchased as ready-to-use formulation presents a strong case for the introduction of Aprokam in hospitals where purchased PFS is still used.

### Study limitations

There are a number of limitations related to the study methodology that may influence the results of this budget impact analysis, and thus should be taken into consideration when interpreting the cost estimates. Firstly, the key inputs of the analysis are resource use data provided by pharmacists, surgeons, and theatre nurses who responded to the survey. Therefore, these inputs may not be applicable to all hospitals and may vary should more robust sources be identified. Changes in these inputs are likely to have an impact on the results and their interpretation.

Due to lack of data, it was not possible to consider complications due to dilution errors and contamination, in terms of management costs (e.g. additional hospitalization days) and their long-term consequences. For example, dilution errors during the preparation of PFS have been linked to higher risks of POE, IGS and TASS [13, 17]. Furthermore, the model does not capture wastage related to the different cefuroxime preparations. It is possible that ready-to-use purchased PFS would lead to higher wastage due to their shorter shelf life and their requirement of refrigeration. It should be noted that not considering complications and wastage is in favour of the comparators and can be assumed to be a conservative approach. In addition, the risk of needlestick injuries could not be assessed for Aprokam and prepared PFS, which both require using needles during their preparation.

Finally, the analysis was conducted on a hypothetical cohort of 1000 cataract surgeries in the starting year, followed by a 5.2 % annual increase in the number of surgeries. This setting is a key driver of the total costs reported in this analysis over the considered time horizon, although it is unlikely to impact the cost per surgery.

## Conclusions

The lower overall total direct costs with Aprokam compared with purchased PFS presents a strong argument for the adoption of Aprokam in clinical settings where unlicensed purchased PFS is still administered. Aprokam presents with cost-savings, and increased liability coverage compared with purchased PFS. Further, there is also the assumption that the Aprokam is less likely to be associated with dilution errors and contaminations, although these claims have not been empirically demonstrated. In NHS hospitals where patients are still receiving unlicensed prepared PFS, hospital managers and local payers should strongly consider allocating additional financial resources to achieve increased liability coverage with Aprokam.

## Abbreviations

BIM: Budget impact mode
ESCRS: European Society of Cataract and Refractive Surgery
GB: Great Britain
HPLC: High performance liquid chromatography
IGS: Irvine gass syndrome
NaCl: Sodium chloride
N/A: Not applicable
NHS: National Health Service
PFS: Pre-filled syringes
POE: Postoperative endophthalmitis
PSS: Personal Social Services
QA: Quality assurance
SPC: Summary of product characteristics
TASS: Toxic anterior segment syndrome
UK: United Kingdom
UV-vis: Ultraviolet-visible spectroscopy

## References

[1] Barry P, Cordoves L, Gardner S (2013). ESCRS guidelines for prevention and treatment of endophthalmitis following cataract surgery : data, dilemmas and conclusions.

[2] Suto C, Morinaga M, Yagi T, Tsuji C, Toshida H (2012). Conjunctival sac bacterial flora isolated prior to cataract surgery. Infect Drug Resist.

[3] The Royal College of Ophthalmologists (2007). Managing an outbreak of postoperative endophthalmitis.

[4] Kamalarajah S, Silvestri G, Sharma N, Khan A, Foot B, Ling R (2004). Surveillance of endophthalmitis following cataract surgery in the UK. Eye (Lond).

[5] ESCRS Endophthalmitis Study Group (2007). Prophylaxis of postoperative endophthalmitis following cataract surgery: results of the ESCRS multicenter study and identification of risk factors. J Cataract Refract Surg.

[6] The Royal College of Ophthalmologists (2010). Cataract surgery guidelines.

[7] Barry P (2014). Adoption of intracameral antibiotic prophylaxis of endophthalmitis following cataract surgery: update on the ESCRS Endophthalmitis Study. J Cataract Refract Surg.

[8] NHS National Patient Safety Agency (2007). Risk assessment tool for the preparation and administration of injectable medicines in clinical areas.

[9] Laboratoires Théa (2013). Aprokam summary of product characteristics.

[10] Delyfer MN, Rougier MB, Leoni S, Zhang Q, Dalbon F, Colin J (2011). Ocular toxicity after intracameral injection of very high doses of cefuroxime during cataract surgery. J Cataract Refract Surg.

[11] Olavi P (2012). Ocular toxicity in cataract surgery because of inaccurate preparation and erroneous use of 50 mg/ml intracameral cefuroxime. Acta Ophthalmol.

[12] ASHP (2014). ASHP guidelines on compounding sterile preparations. Am J Health Syst Pharm.

[13] Lockington D, Flowers H, Young D, Yorston D (2010). Assessing the accuracy of intracameral antibiotic preparation for use in cataract surgery. J Cataract Refract Surg.

[14] Ciftci S, Ciftci L, Dag U (2014). Hemorrhagic retinal infarction due to inadvertent overdose of cefuroxime in cases of complicated cataract surgery: retrospective case series. Am J Ophthalmol.

[15] Qureshi F, Clark D (2011). Macular infarction after inadvertent intracameral cefuroxime. J Cataract Refract Surg.

[16] Yoeruek E, Spitzer MS, Saygili O, Tatar O, Biedermann T, Yoeruek E (2008). Comparison of in vitro safety profiles of vancomycin and cefuroxime on human corneal endothelial cells for intracameral use. J Cataract Refract Surg.

[17] Buyukyildiz HZ, Gulkilik G, Kumcuoglu YZ (2010). Early serous macular detachment after phacoemulsification surgery. J Cataract Refract Surg.

[18] Cakir M, Imamoglu S, Cekic O, Bozkurt E, Alagoz N, Oksuz L (2009). An outbreak of early-onset endophthalmitis caused by Fusarium species following cataract surgery. Curr Eye Res.

[19] Sharifi E, Porco TC, Naseri A (2009). Cost-effectiveness analysis of intracameral cefuroxime use for prophylaxis of endophthalmitis after cataract surgery. Ophthalmology.

[20] Linertova R, Abreu-Gonzalez R, Garcia-Perez L, Alonso-Plasencia M, Cordoves-Dorta LM, Abreu-Reyes JA (2014). Intracameral cefuroxime and moxifloxacin used as endophthalmitis prophylaxis after cataract surgery: systematic review of effectiveness and cost-effectiveness. Clin Ophthalmol.

[21] O’hEineachain R (2014). Femtosecond laser-assisted cataract surgery shows benefits.

[22] Royal Pharmaceutical Society (2009). Professional standards and guidance for the sale and supply of medicines.

[23] WHO (2014). Report for WHO on findings of a review of existing guidance/advisory documents on how medicines should be administered to children, including general instructions on compounding preparations and manipulation of adult dosage forms.

[24] Department of Health (2014). PCT capital accounting manual: depreciation policy for PCTs.

[25] Théa L (2014). Internal communication.

[26] NHS. Unlicensed medicines policy—East London NHS foundation trust. 2014 [http://www.eastlondon.nhs.uk/About-Us/Freedom-of-Information/Trust-Policies-and-Procedure/Medicine-Policies/Unlicensed-Medicines-Policy.pdf].

[27] AAO (2011). Cataract in the adult eye PPP—2011.

[28] BNF (2014). British national formulary 67.

[29] NHS (2014). Agenda for change—pay rates.

[30] PSSRU (2013). Unit costs of health and social care 2013.

[31] Oncall medical supplies (2014). Pharmacy fridges.

[32] Oncall medical supplies (2014). Laboratory fridges, fridge-freezeers.

[33] NHS (2014). General information about pay in the NHS.

[34] Oncall medical supplies (2013). Syringes.

[35] Trust SEF (2011). Items recommended for standardisation in the trust.

[36] The Medical World Experience (2014). Connector (sterile) rapid fill × 50 (BAXA).

[37] Sigma-Aldrich (2014). Syringe pressure caps.

[38] BNF. Searching British national formulary for sodium chloride solution. 2014 [http://www.evidence.nhs.uk/formulary/bnf/current#Search?q=sodiumchloridesolution].

[39] Oncall medical supplies (2013). PM3715 premier clearview sterilisation pouches 133 × 254mm self-seal box of 200.

[40] Cleatech LLC (2014). Benchtop vertical laminar flow hoods - CleaBench™.

[41] Marshall Scientific (2014). Waters 996 HPLC photo diode array detector.

[42] Edmund Optics (2014). Manual mini-chrom monochromators.

[43] Buck scientific (2014). Buck M530 quick-scan infrared spectrophotometer.

[44] Cream Supplies (2014). Glass test tube with white cap (16 mm × 150 mm).

[45] Sigma-Aldrich (2014). Hanna pH meter model HI 9124.

[46] LabX (2014). Osmometers listings.

[47] Oncall medical supplies (2013). Incubator.

